# The Anti-Cytokine Storm Activity of Quercetin Zinc and Vitamin C Complex

**DOI:** 10.1155/2022/1575605

**Published:** 2022-06-08

**Authors:** Hayder B. Sahib, Omer Abid Kathum, Rafal Shakeeb Alanee, Rehab A. M Jawad, Ahmed Majeed Al-Shammari

**Affiliations:** ^1^Al-Nahrain University, College of Pharmacy, Pharmacology Department, Baghdad, Iraq; ^2^Al-Nahrain University, Biotechnology Research Center, Baghdad, Iraq; ^3^Ministry of Health, Kimadia, Iraq; ^4^Experimental Therapy Department, Iraqi Center for Cancer and Medical Genetic Research, Mustansiriyah University, Baghdad, Iraq

## Abstract

Cytokine storm is one of the causative deaths in a patient with severe acute respiratory syndrome. This study aimed at evaluating the prophylaxis effect of quercetin complexes with zinc and buffered ascorbic acid upon cytokine storm induction in mice and identifying the complex's acute toxicity. Mice were randomly divided into three groups: group A, control group, received 0.9% normal saline; group B received 100 mg/kg of the complex one hour before lipopolysaccharide (LPS) administration; and group C received the LPS IP 5 mg/kg. Then, levels of interleukin 1 and interleukin 6 were measured in the serum, and lung and kidney tissues were investigated for any changes that may have happened. Thirty mice were used to investigate the acute toxicity; mice were distributed into six groups: one control group and five treated groups; then several serial dilutions from the complex have been prepared for different concentrations from 5 g/kg to 0.312 g/kg. The animals were observed for 14 days. The LD_50_ was deduced by the straight-line equation calculated from the dose-response curve. The results in this study showed that group A had no significant tissue change. LPS group C showed tissue damage in the lung and kidney, which significantly prevented by the pretreated complex in group B. Moreover, the complex's acute toxicity value (LD_50_) was 655 mg/kg. In conclusion, the complex has significantly ameliorated LPS-induced acute lung and kidney injury, largely through suppression of inflammation; the large lethal dose value may make the complex have a promising therapeutic effect in the prevention of cytokine storm.

## 1. Introduction

The severity of the COVID-19 pandemic and the health complication that may occur upon a patient's infection with the absence of a curable drug was the trigger to find an agent(s) targeting the virus or its sequels. A cytokine storm, also called hypertyrosinemia, is a response in humans in which proinflammatory signaling occurs in an uncontrolled manner. The sudden release of cytokines in large quantities can cause organ failure and death [1]. Cytokine storm (CS) is characterized by a clinical presentation of overwhelming systemic inflammation, hemodynamic instability, and multiorgan failure, and if left untreated, it leads to death. The CS clinical findings are attributed to the action of the proinflammatory cytokines such as IL-1, IL-6, IL-18, IFN-*γ*, and TNF-*α*. Lung injury is one consequence of the cytokine storm that can progress into acute lung injury [2]. Anti-inflammatory modulators hold a huge potential by targeting the cytokine storm effect, which may be very much valuable in lowering COVID-19 patients' morbidity and mortality [3].

**Figure 1:**
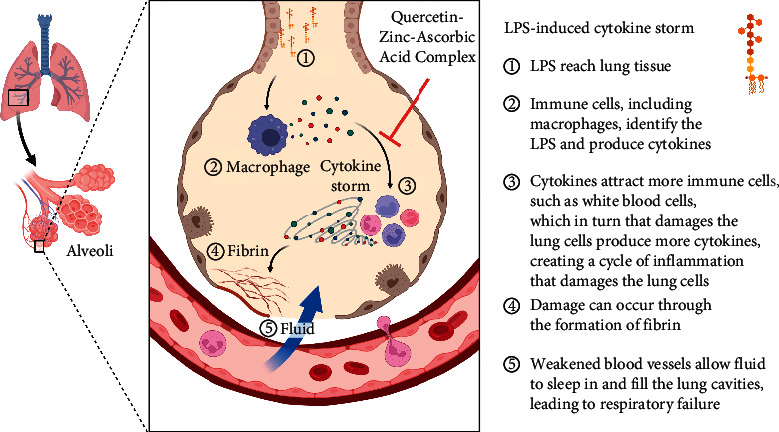
Proposed mechanism for LPS-induced cytokine storm and the proposed interference of the novel complex.

Quercetin is found in many fruits. It has a bitter flavor and is used as a dietary supplement, beverages, and foods; quercetin has a potent antioxidant effect [4], and this effect may be due to the polyphenolic chemical substructure that had been shown by Wang and his coworkers [5]. Quercetin had many pharmacological effects; one showed a robust effect on human breast cancer cell lines [6]. Moreover, quercetin might exert its anti-inflammatory effect through negatively modulating proinflammatory factors, such as IL-6 [7]. Natural compounds hold promise as antiviral agents [8]. Zinc and vitamin C are well-known antioxidants that are used to treat chronic inflammation that may arise from oxidative stress and lead to many chronic illnesses [9]. Zinc is one of the important metal oxides nowadays used clinically for many diseases, and it is considered in drug delivery systems because of its easy formulation, less cost, high safety, and high drug-loading capability. Moreover, zinc exhibits a wide range of antimicrobial activity and anticancer therapeutic effects. Zinc safety was tested, thereby showing that it is one of the safest drugs to be used [10]. Zinc supplementation showed an important role in pulmonary diseases. Zinc showed a significant potent antioxidant activity [11], which was one reason behind choosing it to reduce the incidence and severity of cytokine storm with quercetin and vitamin C, and this work evaluated Quercetin Zinc and Vitamin C Complex activity as an anticytokine storm that may occur during COVID-19 infection as in Figure 1.

## 2. Materials and Methods

### 2.1. Experimental Animal

Albino mice of 20–25 g were used in the experiments. The animals were obtained from the Animal House Facility, National Center for Drug Quality Control and Research in Iraq and kept at 28–30°C. All the animals were allowed free access to food and tap water. The experiments were approved by the Animal Ethical Committee at Al-Nahrain University, College of Pharmacy, under issue number PH-Nah 4.

### 2.2. LPS-Induced Cytokine Storm Model

Mice were fasted overnight; LPS (5 mg/kg body weight; Sigma, St. Louis, MO, USA) was injected intraperitoneally into male mice. The animals were divided randomly into three groups. Group A, the control group, received 300 *μ*l of 0.9% normal saline. In group B, animals were given a single dose of the complex (100 mg/kg, IP) one hour before LPS injection. Group C received LPS only. After 24 h, mice were anesthetized by diethyl ether before blood was collected by jugular vein puncture and centrifuged for 15 minutes at 5000 rpm. Serum was collected and kept under −20°c. Then, IL-1 and IL-6 were measured in all three groups [12].

### 2.3. Measurement of Cytokine Levels (IL-1 and IL-6) in Mice

This study evaluated IL-1 and IL-6 in mice through ELISA kits (CUSABIO, USA); all reagents, samples, and standards were prepared according to the manufacturer's protocols. Here, 100 *μ*l of samples were added to each well and incubated for 2 hours at 37°C, then 100 *μ*l of Biotin antibody (1X) was added to each well with incubation for 1 hour at 37°C, the samples were aspirated and washed three times, and 100 *μ*l of HRP-avidin (1X) was added to each well with incubation of 1 hour at 37°C. Again, the samples were subjected to aspiration and washed five times. After that, 90 *μ*l of the TBM substrate was added to the wells to incubate them for 15–30 minutes at 37°C in the dark. Finally, we added 50 *μ*l of stop solution to each well to stop the reaction and read them at 450 nm within 5 minutes.

### 2.4. Dose Killing 50% of Tested Animals (LD_50_)

This study used thirty male Swiss albino mice weighing 20–25 g to measure acute toxicity. All animals were equally and randomly distributed into six groups: one control group and five treated groups. The researchers followed the code of animal ethics of Al-Nahrain University, College of Pharmacy. The animals were allowed to adapt to the laboratory conditions for seven days before the experiment. The control group received 0.9 normal saline IP, while each treated group received the complex IP. The dose had been prepared by dispersing 10 g of the complex in 10 ml volume of 0.9% normal saline; then, serial dilution of five different doses was prepared; the doses started from 5 g/kg to 0.312 g/kg. The animals were observed continuously for the first 4 h and then every 24 h for the following 48 h after administering the complex [13]. Animals were observed for any death or changes in general behavior and other physiological activities. The LD_50_ was deduced by the straight-line equation calculated from the dose-response curve *Y*=17.103*∗*Lin(*X*)+57.33 was 655*mg*/*kg*.

### 2.5. Histopathology

The tissue had been kept in 10% of buffered formalin. The tissue was dehydrated, embedded, cleared, and infiltrated with the embedding material. After that, the tissue samples were placed into molds and waxed until they became hard. Later, tissues were sectioned into 4 *μ*m sections by using a microtome. Hematoxylin and eosin (H&E) stain was used to stain the nuclei to blue because of its high affinity to nucleic acids in the cell nucleus, while eosin, an acidic dye, stained the cytoplasm.

## 3. Results and Discussion

**Figure 2:**
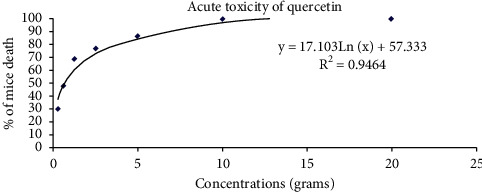
The dose-response curve of the quercetin complex against five groups of randomly distributed mice.

COVID-19 that invaded the world caused a challenge for all the healthcare systems globally and significant challenge to the scientists. This study aimed at investigating the capability of the complex to restrict the cytokine storm resulted from LPS induction; however, the complex toxicity had not been tested before, and that is why, it was crucial to identify the dose that the researchers should start with (Hayder et al., 2014).  Figure 2 shows the dose-response curve for the complex deduced from serial doses administered to the mice groups. The doses and the mice death rate were as follows: 5 g/kg, 2.5 g/kg, 1.25 g/kg, 0.625 g/kg, and 0.312 g/kg killing 87%, 77%, 69%, 48%, and 30%, respectively. LD_50_ had been deduced from the straight-line equation showed in Figure 2, and it was 566 mg/kg. The researchers chose 100 mg/kg as the dose to start with.

Table 1 shows that the novel complex was able to suppress cytokine storm through inhibition of IL-1 and IL-2.

The researcher's team of this study tested a complex of quercetin, zinc, and vitamin C to investigate its capability to prevent cytokine storms [14]. The results in this study showed that IL-6 and IL-1 levels for group C (LPS-administered group) increased significantly (*p* < 0.05) when compared with those of the control group (group A). In contrast, IL-6 and IL-1 levels in group B (pretreated with quercetin complex) showed no significant differences (*p* < 0.05) compared to group A. However, there were significant differences between groups B and C (*p* < 0.05). The results in group C may be attributed to the ability of LPS to activate toll-like receptor 4 (TLR4) that, in turn, leads to activate the nuclear factor kappa light chain kappa factor (NF-*ҡ*B) and MAPK kinase pathway [15]; subsequently, they lead to the expression of many proinflammatory cytokines and a significant increase in the production of reactive oxygen species (ROS) and synthesis of nitric oxide (NO) and prostaglandin E2 (PGE2) [16]. Moreover, activation of the nude-like receptor (NLR), Janus kinases (JAKs)/signal transducer, transcription proteins, and JAK-STAT inflammatory pathways lead to elevated levels of IL-1 and IL-6, thereby cytokine storm produced [17]. On the other hand, the significant reduction in IL-1 and IL-6 levels in group B (a pretreated group with the complex) was maybe due to the antiinflammatory, antioxidant, and immune-modulating activities that this complex had [18]. Moreover, quercetin inhibits the production of inflammation-producing enzymes, e.g., cyclooxygenase (COX) and lipo-oxygenase (LOX), which yield prostaglandin E2 and thromboxane A2 that may exacerbate cytokine storm as well [19, 20]. Scientific evidence approved that quercetin prevents phosphorylation of STAT, which may block the JAK-STAT inflammatory pathway responsible for signaling most cytokines [21, 22].

Other findings suggested that zinc plays a substantial role in the development of metabolic syndrome, regulates cytokine expression, and may have a role in suppressing inflammation. Zinc is necessary to reinforce antioxidant enzymes that scavenge reactive oxygen species, thereby reducing oxidative stress [17]. At the same time, vitamin C has correlated with insufficient phosphorylation of signal transducers and activators of transcription (STATs), which represent a crucial signaling process of cytokines [17].

**Figure 3:**
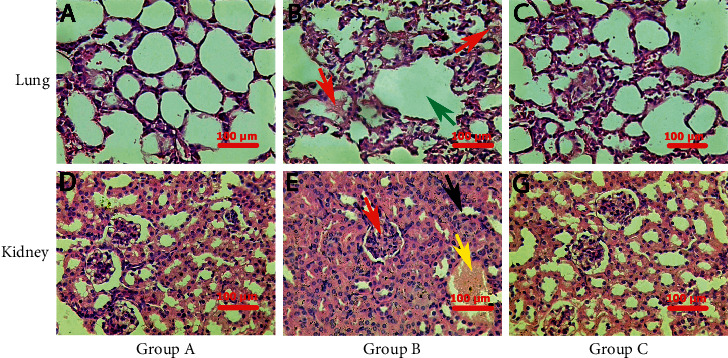
Histopathological findings in the lung and kidney tissue (H&E). (a) Lung tissue section treated with normal saline 0.9% as control showed normal histology of the lung consisting of alveoli surrounded by normal alveolar septae and no inflammatory cell infiltration. (b) Lung section of LPS-injected group C showed alveolar hemorrhage and pretentious material (red arrow), interstitial inflammatory cell infiltration, and diffuse alveolar damage (green arrow) (score 3 tissue damage). (c) Lung tissue section of LPS-injected and quercetin complex-treated group B showed reduced inflammatory cell infiltration in some areas and focal dispersed destruction of alveolar (score 1 tissue damage). (d) The tissue section of the kidney treated with normal saline 0.9% of the control group (group A) showed normal histological appearance, consisting of glomeruli, proximal and distal convoluted tubules, and no interstitial inflammatory cell infiltration. (e) Renal section of LPS-injected group C showed dilated glomerular capillaries (red arrow), congested blood vessels, damage of renal tubules with the presence of hyaline cast inside renal tubules (yellow arrow), and focal inflammatory cell infiltration (black arrow) (score 4 tissue damage). (f) Kidney tissue section of LPS-injected and quercetin complex-treated group B showed only mild degenerative changes of epithelial renal tubules (score 1 tissue damage).

Histological findings further confirmed the results. The lung tissue section treated with normal saline 0.9% as control showed normal histology of the lung consisting of the alveoli surrounded by normal alveolar septae and no inflammatory cell infiltration (Figure 3(a)). At the same time, LPS-injected group C (cytokine storm model group) showed alveolar hemorrhage and pretentious material, interstitial inflammatory cell infiltration, and diffuse alveolar damage, ranking as score 3 tissue damage (Figure 3(c)). In contrast, quercetin complex-treated group B showed reduced inflammatory cell infiltration in some areas and focal dispersed destruction of the alveoli, ranking score 1 tissue damage, proving that the novel complex can reduce the tissue damage induced by the cytokine storm (Figure 3(b)).

Kidney is the second most important organ in the body that is affected by the cytokine storm. The tissue section of the kidney from group A treated with normal saline 0.9% as control showed normal histological appearance, consisting of glomeruli, proximal and distal convoluted tubules, and no interstitial inflammatory cell infiltration (Figure 3(d)). In contrast, LPS-injected group C showed dilation and congestion of glomerular capillaries and blood vessels, damage of renal tubules with the presence of hyaline cast inside renal tubules and focal inflammatory cells. The tissue damage is scored 4 (Figure 3(e)). Quercetin complex administration reduced the histological changes to mild degenerative changes of renal epithelial tubules. Moreover, it reduces tissue damage score to 1 only. These findings support the researcher's claims of the effectiveness of the complex.

However, in group C (LPS-treated group), there were significant histopathological changes in the lung tissue, while the inflammatory damage included an intra-alveolar hemorrhage with proteinaceous material, interstitial inflammatory cell infiltration, and diffuse alveolar damage, ranking score 3 damage [23]. During lung injury, the proposed pathogenesis after LPS administration is mediated by the TLR/NF-*κ*B pathway and other inflammatory pathways that accelerate the release of a huge amount of proinflammatory cytokines and neutrophil sequestration and activation. Moreover, epithelial damage is caused by oxidants, proteases, leukotrienes, and other proinflammatory molecules released by neutrophils, such as platelet-activating factors. When epithelial integrity is compromised, normal epithelial fluid transport is disrupted, resulting in alveolar destruction and a decrease in surfactant synthesis [22]. Renal tissue showed a score of 4 damage characterized by acute destruction of renal tubules with the presence of a hyaline cast and focal inflammatory cell infiltration. Moreover, there were blood vessel congestion and dilatation, all scored as number 4 damage. This damage is mediated by the formation of cytotoxic ROS that activates the NF-*κ*B signal pathway which is associated with inflammation and renal injury that leads to renal failure [23]. The pathological changes that occur in the tissues of group B (pretreated with the quercetin complex) confirm their effectiveness to decrease the degenerative area, inflammatory cell infiltration, and improvement in the score of damage in comparison with group C.

## 4. Conclusion

**Figure 4:**
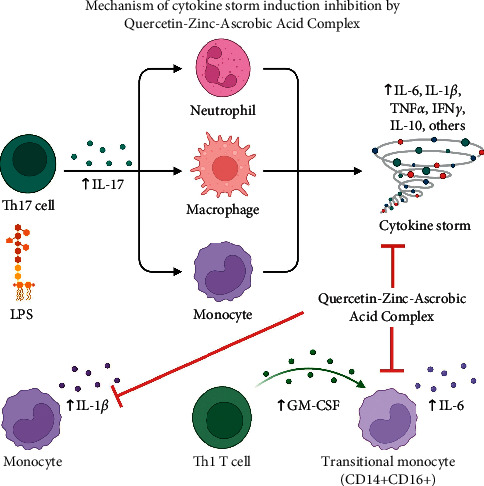
The quercetin complex has a protective effect against tissue damage induced by a cytokine storm in mice. This may be attributed to the quercetin complex being responsible for regulating inflammation through downregulating IL-1 and IL-2. This figure was created with the help of BioRender.com.

The study concluded that quercetin complex has a protective effect against tissue damage induced by a cytokine storm in mice. This result may be attributed to the quercetin complex being responsible for regulating inflammation through downregulating IL-1 and IL-2, as summarized in Figure 4, and this may make this complex have a great therapeutic value against cytokine storm.

## Figures and Tables

**Table 1:** Interleukin 1 and interleukin 6 levels in mice.

Group A/N = 8, IL-1 and IL-6		Group B/N = 8, IL-1 and IL-6		Group C/N = 8, IL-1 and IL-6	
6	4	8	12	78	44
4	4	8	10	76	47
4	4	12	11	79	55
4	5	10	9	73	56
4	3	11	9	75	43
5	3	11	8	73	48
4	2	9	11	78	55
3	6	9	7	77	44

*N* = number of mice; Group A, containing 8 mice, receives nothing but 0.9% NS, which is used to dissolve the compound. Group B contains 8 mice injected with lipopolysaccharides (LPS) and the complex. Group C received LPS only.

## Data Availability

The experimental data used to support the findings of this study may be released upon application to the corresponding author, who can be contacted at [haider_bahaa@yahoo.com].
